# Taxogenomics of the Genus *Cyclobacterium*: *Cyclobacterium xiamenense* and *Cyclobacterium halophilum* as Synonyms and Description of *Cyclobacterium plantarum* sp. nov.

**DOI:** 10.3390/microorganisms8040610

**Published:** 2020-04-23

**Authors:** Azadeh Shahinpei, Mohammad Ali Amoozegar, Leila Mirfeizi, Mahdi Moshtaghi Nikou, Antonio Ventosa, Cristina Sánchez-Porro

**Affiliations:** 1 Extremophiles Laboratory, Department of Microbiology, Faculty of Biology and Center of Excellence in Phylogeny of Living Organisms, College of Science, University of Tehran, 1417414418 Tehran, Iran; a.shahinpei@yahoo.com (A.S.); amoozegar@ut.ac.ir (M.A.A.); leilamirfeizi@ymail.com (L.M.); 2 Microorganisms Bank, Iranian Biological Resource Centre (IBRC), ACECR, 1551916111 Tehran, Iran; mahdi.moshtaghi.nico@gmail.com; 3 Department of Microbiology and Parasitology, Faculty of Pharmacy, University of Sevilla, 41012 Sevilla, Spain; sanpor@us.es

**Keywords:** *Cyclobacterium*, *Cyclobacterium xiamenense*, *Cyclobacterium halophilum*, *Cyclobacterium plantarum*, taxogenomics, bacterial taxonomy, halophilic bacteria, new species

## Abstract

The genus *Cyclobacterium* belongs to the phylum *Bacteroidetes* and includes eight species. Our study, based on the genomic parameters in silico DNA–DNA hybridization (GGDC), average nucleotide identity (OrthoANI), and average amino acid identity (AAI), confirmed that all current species of *Cyclobacterium* belong to this genus and constitute a coherent phylogenomic group, but with species forming two separate branches. In addition, the genome-based analyses revealed that *Cyclobacterium xiamenense* and *Cyclobacterium halophilum* are members of the same species. Besides, we carried out a taxonomic characterization of the new strain GBPx2^T^, isolated from the halophytic plant *Salicornia* sp. Analysis of its 16S rRNA gene sequence showed the highest sequence similarity (97.5%) to *Cyclobacterium lianum* HY9^T^. Percentages of GGDC and OrthoANI between strain GBPx2^T^ and species of the genus *Cyclobacterium* were lower than the threshold value for species delineation. The DNA G+C content was 43.0 mol%. The polar lipids included phosphatidylethanolamine as well as one unidentified phospholipid and four unidentified lipids, and its major cellular fatty acids were iso-C_15:0_ and summed feature 3 (C_16:1_*ω7c* and/or iso-C_15:0_ 2-OH). The only quinone present was menaquinone 7. Based on a combination of phenotypic, chemotaxonomic, and phylogenomic features, the GBPx2^T^ strain represents a novel species of the genus *Cyclobacterium*, for which the name *Cyclobacterium plantarum* sp. nov. is proposed. The type strain of *Cyclobacterium plantarum* is GBPx2^T^ (= IBRC-M 10634^T^ = LMG 28551^T^).

## 1. Introduction

The genus *Cyclobacterium* is the type genus of the family *Cyclobacteriaceae*, order *Cytophagales*, within the class *Cytophagia*, in the phylum *Bacteroidetes* [1]. This genus was originally described by Raj and Maloy [2] and has been emended three times [3,4,5]. *Cyclobacterium marinum* (type species) was initially described as *Microcyclus marinus* (referring to its vibrioid bacterial cell morphology that exhibits a closed ring-like morphology) [6], and was reclassified as *Flectobacillus marinus* [7]. Recently, the genus *Cyclobacterium* has been established as a separate genus and species from the genus *Flectobacillus*, which includes its freshwater counterparts [2]. The genus *Cyclobacterium* comprises eight species with valid published names: *Cyclobacterium marinum* (type species) [2], *Cyclobacterium amurskyense* [8], *Cyclobacterium lianum* [3], *Cyclobacterium qasimii* [9], *Cyclobacterium caenipelagi* [4], *Cyclobacterium jeungdonense* [10], *Cyclobacterium xiamemense* [5], and *Cyclobacterium halophilum* [11]. There is also another species, “*Cyclobacerium sediminis*”, which was described in 2017, but its name has not been validated to date [12]. These species have been isolated from different saline habitats such as seawater, marine sediments, soil from solar salterns, aggregates of the alga *Chlorella autotrophica*, or from sea cucumber [2,3,4,5,8,9,10,11]. Furthermore, 16S rRNA gene sequences related to this genus have also been reported by culture-dependent and/or culture-independent studies in different habitats such as a soda saline crater lake [13], microbial mats from Antarctic lakes [14], or from an alkaline, cold habitat in Greenland [15]. The species of this genus include Gram-stain-negative, curved ring-like or horseshoe-shaped bacteria. These species are non-flagellated, non-motile, aerobic, and heterotrophic, and their colonies are pigmented pink to orange/red. The major or sole respiratory quinone is MK-7. They are psychrotolerant to mesophilic and halotolerant to moderately halophilic. The G+C content of their DNA ranges from 33.7 to 48.1 mol% and their major cellular fatty acids (>10%) are iso-C_15:0_ and summed feature 3 (comprising C_16:1_ω*7c* and/or C_16:1_*ω6c*) [2,3,4,5,8,9,10,11].

In 2010, strain GBPx2^T^ was isolated from *Salicornia* sp., a halophytic plant, in the Gomishan wetland, Iran. This strain was affiliated to the genus *Cyclobacterium* but differed in some phenotypic and phylogenetic features from the *Cyclobacterium* species. We propose that it represents a novel species of the genus *Cyclobacterium.* Genome-based analysis was performed, using the genome sequences of the type strains of the species of *Cyclobacterium* and those of species of the family *Cyclobacteriaceae* available in databases, in order to carry out a taxogenomic study and determine in detail the phylogenomic relationships among species of the genus *Cyclobacterium* and other members of the family.

## 2. Materials and Methods

### 2.1. Bacterial Strains

Strain GBPx2^T^ was isolated from *Salicornia* sp., a halophytic plant of the Gomishan wetland, an alkaline, thalassohaline, coastal-marine wetland located along the eastern shore of the Caspian Sea in Iran. The wetland water contains 30–50 g dissolved salts per liter, and the average pH is 8.8 [16]. The geographic coordinates of the sampling location were 37° 03ʹ 64.2ʺ N 054° 01ʹ 90.4ʺ E. Plants were sampled from southeastern the wetland in November 2010. The novel strain was isolated from a halophytic plant by using serial dilutions: 10 g of the sample was weighed and added to 90 mL of sterile 3.0% (w/v) NaCl solution. Serial dilutions were plated on Marine Agar 2216 (MA; Difco) and incubated at 30 °C for two weeks. The colony of the strain was subsequently purified three times by plating on the same medium. It was maintained on the same medium and also at −80 °C in MA medium without agar and supplemented with 37.8% (w/v) glycerol.

The type strains of three *Cyclobacterium* species were obtained from the Iranian Biological Resource Center (IBRC) and used as reference strains. These were *Cyclobacterium lianum* IBRC-M 10422^T^, *Cyclobacterium jeungdonense* IBRC-M 11102^T^, and *Cyclobacterium halophilum* IBRC-M 10761^T^. They were cultured following the recommendations of the culture collection.

### 2.2. Taxophylogenomic Characterization

#### 2.2.1. DNA Extraction, Purification, and Sequencing

DNA was extracted following the protocol of Marmur [17]. The DNA quality was checked by (0.8%) agarose gel electrophoresis. The quantification of the extracted DNA was determined by spectrophotometry (DeNovix DS-11 FX, DeNovix Technologies, Wilmington, DA, USA) and fluorometry (Qubit 3.0 Fluorometer, Thermofisher Scientific, Waltham, MA, USA). PCR products were purified using the commercial kit MEGAquick-spinTM Plus (INtRON Biotecnology, Labotaq, Sevilla, Spain). Sequencing of the 16S rRNA PCR products was carried out by Macrogene (Sangdaewon-dong, Gyeonggi-do, South Korea) using the Sanger method and the primers 16F27 (5′-AGAGTTTGATCMTGGCTCAG-3′), 16R343 (5′-ACTGCTGCCTCCCGTA-3′), 16F530 (5′-GTGCCAGCAGCCGCGG-3′), and 16R1488 (5′-CGGTTACCTTGTTAGGACTTCACC-3′) [18], and the genome of strain GBPx2^T^ was sequenced using the Illumina NovaSeq 6000 platform (Novogene Europe, Cambridge, UK).

#### 2.2.2. Phylogenetic Analysis Based on 16S rRNA Gene Sequence Comparison

**Figure 1:**
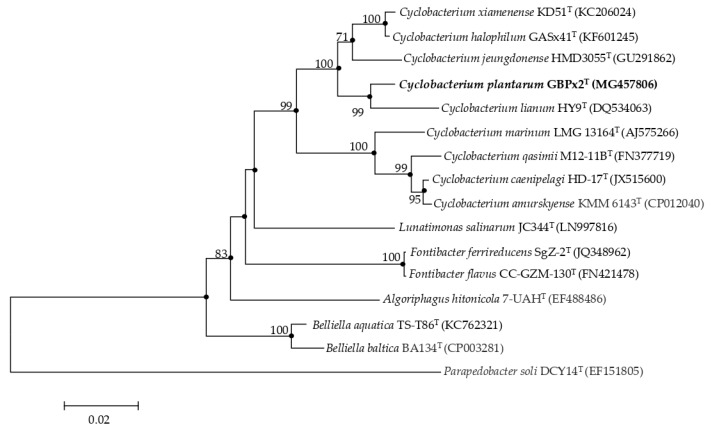
Maximum-likelihood phylogenetic tree based on the 16S rRNA gene sequence comparison, showing the relationships between strain GBPx2^T^ and members of the family *Cyclobacteriaceae*. Filled circles indicate nodes that were also obtained in trees based on minimum-evolution and maximum-likelihood algorithms. Bootstrap values (for 1000 replicates) over 70% are shown at the nodes. The sequence accession numbers are shown in parenthesis. Bar, 2% estimated sequence divergence. The sequence of *Parapedobacter soli* DCY14^T^ (EF151805) was used as outgroup.

The partial 16S rRNA gene was amplified using the universal primer pairs 16F27 and 16R1488 [19]. The PCR products were visualized on 1% agarose gel. The forward and reverse sequences were assembled by using Chromas Pro 1.7.7 (Technelysium Pty Ltd., South Brisbane, Australia). The 16S rRNA gene sequence of strain GBPx2^T^ was obtained and used for BLAST searches in GenBank and phylogenetic analysis. The identification of phylogenetic neighbors and calculation of pairwise 16S rRNA gene sequence similarity were achieved using the EzBioCloud server (https://www.ezbiocloud.net/) [20] and the alignments were performed by CLUSTAL-X [21]. Evolutionary distances between aligned 16S rRNA gene sequences of strain GBPx2^T^ with the most closely related type strains were calculated using the Jukes–Cantor model, and phylogenetic trees were reconstructed by the neighbour-joining [22], minimum-evolution [23], and maximum-likelihood [24] methods using the MEGA version 6 program [25]. Bootstrap analysis was carried out to evaluate the tree topology by performing resampling 1000 times [26]. The GenBank/EMBL/DDBJ accession number for the 16S rRNA gene sequence of strain GBPx2^T^ is MG457806. The 16S rRNA gene sequences of the reference type strains used for the phylogenetic comparison were obtained from GenBank database and their accession numbers are shown in Figure 1.

#### 2.2.3. Genome Assembly and Annotation

The novo assembly of the reads of the genome of strain GBPx2^T^ was performed using Spades 3.13.0 [27]. The quality of final contigs was assessed by bioinformatics tools CheckM v1.0.5 [28] and Quast v2.3 [29]. The genome sequence was annotated using the NCBI Prokaryotic Genome Annotation Pipeline (PGAP) [30]. The genome of strain GBPx2^T^ was deposited in GenBank/EMBL/DDBJ under the accession number JAANYN00000000.

#### 2.2.4. Phylogenomic Comparative Analysis

For the phylogenomic comparative analysis we used genomes available from GenBank database. The characteristics of the genomes and their accession numbers of the type strains of species of the genus *Cyclobacterium* are shown in Table 1. The quality of these genome sequences was in accordance with the recommended minimal standards for the use of genome data for the taxonomy of prokaryotes [31]. To determine the core-genome, the Enveomics [32] tool was used. To identify clusters of orthologous genes (OGs), an all-versus-all BLAST search based on protein-coding gene annotated sequences of strain GBPx2^T^ and all type species of the genera included in the family *Cyclobacteriaceae* available in databases was carried out. Those OGs shared among all taxa and present in a single copy per genome were selected. They were aligned with MUSCLE v. 3.8.31 [33] and subsequently concatenated. A maximum-likelihood tree was constructed using FastTree v. 2.1.9 [34] with the JTT replacement matrix [35] under the CAT approximation (single rate for each site) with 20 rate categories. Local support values were estimated with the Shimodaira–Hasegawa test [36].

#### 2.2.5. In Silico DNA–DNA Hybridization (GGDC), Average Nucleotide Identity (ANI), and Average Amino Acid Identity (AAI) 

The genomic parameters of in silico DNA–DNA hybridization (GGDC), average nucleotide identity (OrthoANI), and average amino acid identity (AAI) among strain GBPx2^T^, the type strains of species of the genus *Cyclobacterium*, and the type species of the family *Cyclobacteriaceae* available from databases were determined. GGDC was calculated by the bioinformatic tool Genome-to-Genome Distance Calculator (GGDC version 2.1) available from the Leibniz Institute DSMZ [37]. The OrthoANI was calculated with ChunLab’s Orthologous Average Nucleotide Identity Tool (OAT) [38]. For the estimation of the AAI, the CompareM program (https://github.com/dparks1134/CompareM) was used.

### 2.3. Phenotypic Characterization

Cell morphology and motility were examined using an Olympus BX51 microscope equipped with phase-contrast optics with cells from exponentially growing cultures. Gram staining was performed by the Burke method [39]. Motility was determined by the wet-mount method [39]. Colony morphology was observed on MA agar medium under optimal growth conditions after incubation at 25 °C for two days. To determine the temperature and pH ranges for growth, broth cultures of MA medium were incubated at 0, 4, 10, 15, 20, 25–37 (at intervals of 1.0 °C), 40, and 45 °C and at pH 5–10 at intervals of 0.5 pH units; the buffers sodium acetate/acetic acid (pH 5.0–6.0), Tris/HCl (pH 6.5–8.5), and glycine/sodium hydroxide (pH 9.0–10.0) were added at a concentration of 50 mM. The requirements for NaCl for growth were determined in media containing 1.0, 2.0, 3.0, 4.0, 5.0, 6.0, 7.5, 10.0, 12.5, and 15.0% (w/v) NaCl. Liquid cultures were incubated on a shaking incubator at 150 rpm and growth rates were determined by monitoring the increase in the optical density (OD) at 600 nm (ThermoSpectronics Spectronic 20D+).

Catalase and oxidase tests, nitrate and nitrite reduction, hydrolysis of aesculin, and production of indole and H_2_S were carried out as recommended by Smibert and Krieg [40], using media with 5% (w/v) NaCl. Hydrolysis activity of Tween 20, 40, and 80 was detected as described by Gutiérrez and González [41]. Hydrolysis of gelatin, casein, tyrosine, and starch, and activity for urease and DNase were determined as described by Mata et al. [42]. The anaerobic growth of the strain was tested in the presence of nitrate by adding 0.1% (w/v) KNO_3_ to the medium with 5% (w/v) NaCl in filled stoppered tubes in an anaerobic chamber [43]. Acid production from carbohydrates was tested in unbuffered medium and was determined by measuring the initial and final pH of the medium. The culture was considered positive for acid production if the pH decreased by at least 1 unit. Tests for the utilization of different compounds as the sole source of carbon and energy were performed as recommended by Ventosa et al. [44].

### 2.4. Antimicrobial Susceptibility

Antimicrobial susceptibility tests were performed on Mueller–Hinton agar plus 5% (w/v) marine salts [44] seeded with a bacterial suspension (in 5% [w/v] salts) containing 1.5 × 10^6^ c.f.u. mL^−1^ using discs (HiMedia) impregnated with various antimicrobial compounds. The plates were incubated at 25 °C for 48 h and the inhibition zone was interpreted according to the manufacturer’s manual. The following antimicrobial compounds were used: amoxicillin (30 μg), ampicillin (10 µg), bacitracin (10 µg), carbenicillin (100 μg), cefradine (30 µg), ceftazidime (30 µg), cephalothin (30 µg), chloramphenicol (30 µg), erythromycin (15 µg), gentamicin (10 µg), kanamycin (5 µg), nalidixic acid (30 µg), neomycin (30 µg), nitrofurantoin (300 µg), novobiocin (5 µg), penicillin G (10 U) polymyxin B (300 U), rifampicin (5 μg), streptomycin (10 µg), and tetracycline (30 µg).

### 2.5. Chemotaxonomic Characterization

Cell biomass for fatty acids, isoprenoid quinones, and polar lipids analyses was obtained by cultivation on MA medium at pH 8 and 25 °C. Cells were harvested in the mid-exponential growth phase determined spectrophotometrically with an optical density at 600 nm (OD_600_). The whole-cell fatty acids composition of strain GBPx2^T^ was determined according to the standard protocol of the Microbial Identification System (MIDI, Version 6.1; Identification Library TSBA40 4.1; Microbial ID). Extracts were analyzed using a Hewlett Packard model HP6890A gas chromatograph equipped with a flame-ionization detector as described by Kämpfer and Kroppenstedt [45]. Fatty acids peaks were identified using the TSBA40 database. The polar lipids and respiratory quinones of strain GBPx2^T^ were analyzed as described by Groth et al. [46].

## 3. Results and Discussion

### 3.1. Phylogenetic Analysis Based on 16S rRNA Gene Sequence Comparison

The 16S rRNA gene sequence comparative analysis of strain GBPx2^T^ (1438 nt) showed the highest similarity to *Cyclobacterium lianum* HY9^T^, *Cyclobacterium jeungdonense* HMD3055^T^, *Cyclobacterium xiamenense* KD51^T^, and *Cyclobacterium halophilum* IBRC-M 10761^T^ with 97.5%, 96.7%, 96.2%, and 96.2% sequence similarity, respectively, and values lower than 92.3% with species of other genera, such as *Belliella* or *Fontibacter*. These percentages were obtained by the EzBioCloud tool and indicate that strain GBPx2^T^ is a member of the genus *Cyclobacterium*.

The 16S rRNA gene sequence phylogenetic analysis using the maximum-likelihood algorithm showed the position of the novel strain within the genus *Cyclobacterium* (Figure 1). The phylogenetic position was also confirmed in trees generated using the minimum-evolution and neighbour-joining algorithms.

This phylogenetic tree shows that the genus *Cyclobacterium* is not monophyletic; the species of this genus are grouped into two clearly differentiated branches supported with 100% values of bootstrap. On the one hand, *C. xiamenense* KD51^T^, *C. halophilum* GASx41^T^, *C. jeungdonense* HMD3055^T^, *C. lianum* HY9^T^, and the new isolate GBPx2^T^ appear grouped, and on the other hand *C. marinum* LMG 13164^T^, *C. qasimii* M12-11B^T^, *C. caenipelagi* HD-17^T^, and *C. amurskyense* KMM 6143^T^ are clustered. To determine the relationship between these two clusters, a phylogenomic comparative analysis between them and also with members of other genera of the family *Cyclobacteriaceae* was performed.

### 3.2. Phylogenomic Comparative Analysis

**Figure 2:**
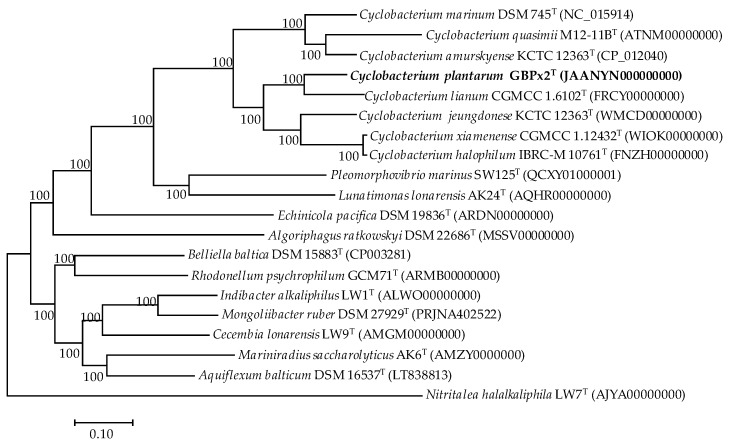
Phylogenomic tree based on the core orthologous translated genes of strain GBPx2^T^, type species of *Cyclobacterium*, and type species of the genera of the family *Cyclobacteriaceae* obtained from the genomes available in databases, based on the maximum-likelihood algorithm. This tree was obtained after the alignment of 1309 shared orthologous single-copy translated genes of these genomes. Bootstrap values higher than 70% are indicated at branch-points. Bar, 0.1 substitutions per amino acid position.

We carried out phylogenomic comparative analysis and obtained the core-genome tree, based on 1309 single-copy translated genes of strain GBPx2^T^, the genomes available for the type strains of *Cyclobacterium* species (Table 1), and the genomes of all type species of the genera of the family *Cyclobacteriaceae* available in databases (Figure 2). This analysis shows that strain GBPx2^T^ constitutes a taxon which is sufficiently different from the other species of *Cyclobacterium* so as to be considered as a new species. Further, as occurred in the phylogenetic tree based on the 16S rRNA, the species of the genus *Cyclobacterium* appeared grouped in two different branches. Finally, this phylogenomic tree showed a close phylogenetic relationship between *Cyclobacterium xiamenense* CGMCC 1.12432^T^ and *Cyclobacterium halophilum* IBRC-M 10761^T^, two species that were described almost simultaneously in 2014 [5,11], and so they were not considered for a comparison between them. Besides, the genomes of these two species are only now available for comparison and the current comparative data show in this study revealed that both are members of the same species.

### 3.3. in silico DNA–DNA Hybridization (GGDC), ANI, and AAI Values

In order to confirm that strain GBPx2^T^ was indeed a new taxon and the relationship between *C. xiamenense* and *C. halophilum* and the two clusters of the genus *Cyclobacterium*, average nucleotide identity (OrthoANI), average amino acid identity (AAI), and in silico DNA–DNA hybridization (GGDC) for the strain GBPx2^T^ and members of the family *Cyclobacteriaceae* were calculated.

GGDC percentages above or equal to 70% indicate that the strains can be assigned to the same species, and values under 70% indicate that the strains belong to different species [47,48,49]. GGDC values were equal or lower than 35% between strain GBPx2^T^ and species of the genus *Cyclobacterium* (Table 2), proving that this strain constitutes a new species. In addition, the GGDC value of 81.6% which was determined between *C. xiamenense* CGMCC 1.12432^T^ and *C. halophilum* IBRC-M 10761^T^, which was higher than the threshold percentage of 70% for species delineation, shows that both species belong to the same taxon [31,37]. With respect to the GGDC values between the other members of this family, all were lower than 70%, showing that all of them can be considered different taxa at the species level.

OrthoANI percentages calculated between strain GBPx2^T^ and species of the genus *Cyclobacterium* ranged from 71.8% to 79.2% (Table 2), lower than the threshold value for species delineation (95%–96%) [31,38,49,50], showing that strain GBPx2^T^ belongs to a different species. Values between 67.2% and 69.9% with the type species of the other genera of the family *Cyclobacteriaceae* were obtained. Further, the OrthoANI value of 97.8% between *C. xiamenense* CGMCC 1.12432^T^ and *C. halophilum* IBRC-M 10761^T^ showed again that both species constituted a single taxon.

An alternative to GGDC and ANI for more distantly related genomes is the AAI. In this case, to confirm that strain GBPx2^T^ and all species of *Cyclobacterium* were well assigned to this genus, the AAI percentages between them were calculated. The AAI values between each other were in the range of 72.2%–97.9% (Table 3). These values were above the threshold considered for species of the same genus (65%) [50,51,52], so we can affirm that all species belong to the genus *Cyclobacterium*. It is remarkable to highlight that AAI values between *C. marinum* DSM 745^T^, *C. qasimii* M12-11B^T^, and *C. amurskyense* KMM 6143^T^ were higher (83.5% to 87.5%) as compared to with other species of *Cyclobacterium* (72.2%–73.5%), and lower than 66.2% with respect to species of the rest of genera of the family *Cyclobacteriaceae*. Similar results were observed in the other group of species of the genus *Cyclobacterium* that appear grouped in the 16S rRNA phylogenetic tree (Figure 1) and also in the core-genome tree (Figure 2). This group included *C. xiamenense* KD51^T^, *C. halophilum* IBRC-M 10761^T^, *C. jeungdonense* HMD3055^T^, *C. lianum* CGMCC 1.6102^T^, and the new isolate GBPx2^T^. AAI values between them ranged from 77.2% to 73.5%. With respect to the other species of the genus *Cyclobacterium* the AAI ranged between 72.2% and 73.5%, and values ranged between 61.6% to 68.1% with regard to the rest of the genera of the family *Cyclobacteriaceae*. All these data showed that the percentages for species of *Cyclobacterium* were always higher than 65% and thus they are members of the same genus, although there was a higher similarity between the respective members of the two phylogroups. Therefore, we conclude that the genus *Cyclobacterium* is monophyletic within the family, but once differentiated, it is divided into two clearly separated groups, as observed previously in both the 16S rRNA and core-genome trees (Figure 1 and Figure 2). On the other hand, the value of 97.9% confirms that *C. xiamenense* and *C. halophilum* are members of the same taxon, as was described by Konstantinidis et al. [51] who established the threshold AAI range of 95%–100% for strains of the same species.

### 3.4. Phenotypic Characterization

Cells of strain GBPx2^T^ were strictly aerobic, non-motile curved rods that were ring-like or horseshoe-shaped, and stained Gram-negative. The colonies were circular, convex with entire margins, translucent, smooth, and pigmented with a light pink color on agar plates. The novel strain was a mesophilic, moderately halophilic, and slightly alkaliphilic bacterium, which grew at a temperature range of 4–40 °C (optimum 25 °C) and a pH range of 6.5–9.0 (optimum pH 8.5) (Table 4). The strain was capable of growing in media with 3%–10% (w/v) NaCl. It grew optimally in the presence of 5% (w/v) NaCl. Strain GBPx2^T^ was catalase- and oxidase-positive. The isolate was sensitive to ceftazidime (30 µg), chloramphenicol (30 µg), erythromycin (15 µg), gentamicin (10 µg), nalidixic acid (30 µg), nitrofurantoin (300 µg), novobiocin (5 µg), rifampicin (5 μg), streptomycin (10 µg), and tetracycline (30 µg), but resistant to amoxicillin (30 μg), ampicillin (10 µg), bacitracin (10 µg), carbenicillin (100 μg), cefradine (30 µg), cephalothin (30 µg), kanamycin (5 µg), neomycin (30 µg), penicillin G (10 U), and polymyxin B (300 U). The detailed physiological and biochemical characteristics of strain GBPx2^T^ as well as its differential features with other related species of the genus *Cyclobacterium* are included in Table 4 and in the new species description. Besides the salinity, temperature, and pH range for growth, other phenotypic features such as the reduction of nitrate, hydrolysis of aesculin and Tween 20, production of acid from some carbohydrates, or the utilization of some compounds permit the differentiation of the new species with respect to related species of *Cyclobacterium*.

### 3.5. Chemotaxonomic Characterization

The cellular fatty acid profile of strain GBPx2^T^ was characterized by the presence of iso-C_15:0_ (26.3%), summed feature 3 (C_16:1_*ω7c* and/or iso-C_15:0_ 2-OH; 23.9%), iso-C_17:0_ 3-OH (12.5%), anteiso-C_15:0_ (12.1%), and iso-C_17:1_*ω9c* (9.6%) as the major fatty acids. The fatty acid profile of the strain was similar to that of the other type strains of species of the genus *Cyclobacterium* (Table 5). However, the percentages of these fatty acids were different from those obtained for other phylogenetically related species.

The polar lipids determined for strain GBPx2^T^ were phosphatidylethanolamine (PE), one unidentified phospholipid (PL), and four unidentified lipids (Supplementary Figure S1). The polar lipids pattern is similar to that of other species in the genus *Cyclobacterium,* except for *Cyclobacterium halophilum,* which has phosphatidylcholine as the major polar lipid [11].

Menaquinone 7 (MK-7) was the only respiratory quinone present in strain GBPx2^T^, which was typically found in members of the genus *Cyclobacterium* [6].

## 4. Conclusions

On the basis of the results of the taxogenomic and polyphasic taxonomic analysis, it is concluded that strain GBPx2^T^ should be considered as a novel species of the genus *Cyclobacterium*, for which the name *Cyclobacterium plantarum* sp. nov. is proposed. We enclose below the taxonomic description of this new species. As a result of the genomic analysis we can conclude that the genus *Cyclobacterium* is a coherent genus within the family *Cyclobacteriaceae*, and that all species currently described are members of the genus, even considering that they constitute two separate phylogenomic clusters. On the other hand, this genome-based study shows that *Cyclobacterium xiamenense* and *Cyclobacterium halophilum* constitute a single species, having priority the name *Cyclobacterium xiamenense* according to the International Code of Nomenclature of Prokaryotes [53]. Thus, *Cyclobacterium halophilum* Shahinpei et al. 2014 should be considered as a later heterotypic synonym of *Cyclobacterium xiamenense* Chen et al. 2014, and in accordance we have included an emended description of the latter species (below).

### 4.1. Description of *Cyclobacterium plantarum* sp. nov.

*Cyclobacterium plantarum (*plan.ta’rum. L. gen. pl. n. *plantarum*, of plants).

Cells are Gram-stain-negative, non-motile, and strictly aerobic curved ring-like or horseshoe-shaped rods with sizes of 0.3–0.5 µm in width and the outer diameter of rings is 0.9–1.9 µm when grown on marine medium under optimal conditions. Colonies are small, circular, convex with entire margins, translucent, and smooth, with a light pink pigmentation. The strain is moderately halophilic and slightly alkaliphilic, growing over a wide range of temperatures from 4 to 40 °C (optimal growth at 25 °C), pH 6.5–9.0 (optimally at pH 8.0) and at 3%–10% (w/v) NaCl (with best growth at 5% [w/v] NaCl). It is positive for catalase and oxidase. Nitrate and nitrite reduction are positive and gas is formed from nitrate. Indole is not produced from tryptophan and H_2_S production is negative. Aesculin and Tween 40 are hydrolyzed, whereas casein, DNA, gelatin, starch, Tween 20, Tween 80, tyrosine, and urea are not. Acid is not produced from D-arabinose, cellobiose, D-galactose, D-glucose, lactose, maltose, melezitose, melibiose, sucrose, raffinose, D-rhamnose, D-ribose, trehalose, or D-xylose. Methyl red and Voges–Proskauer tests are negative. D-arabinose, D-galactose, D-glucose, D-fructose, D-maltose, D-mannitol, D-mannose, D-melibiose, *myo*-inositol, cellobiose, sucrose, D-xylose, ribose, L-alanine, L-ornithine, L-proline, L-serine and L-threonine are utilized as sole source of carbon and energy but L-glutamic acid is not. Polar lipids are phosphatidylethanolamine, one unidentified phospholipid, and four unidentified lipids. The only isoprenoid quinone is MK-7 and the predominant fatty acids are iso-C_15:0_, summed feature 3 (C_16:1_ *ω7c* and/or iso-C_15:0_ 2-OH), iso-C _17:0_ 3-OH, and anteiso-C_15:0_. DNA G+C content of DNA is 43.0 mol% (genome).

The type strain, GBPx2^T^ (= IBRC-M 10634^T^ = LMG 28551^T^), was isolated from *Salicornia* sp., a halophytic plant in the Gomishan wetland, Iran.

The GenBank/EMBL/DDBJ accession number for the 16S rRNA gene sequence and complete genome sequence of the type strain are MG457806 and JAANYN00000000, respectively.

### 4.2. Emended description of *Cyclobacterium xiamenense* Chen et al. 2014

*Cyclobacterium xiamenense* (xia.men.en’se. N. L. neutr. adj. *xiamenense,* of Xiamen, a city in Fujian Province, China where the type strain was isolated).

The description is that of Chen et al. [14], with the following modification: Growth occurs at 1.0%–10% (w/v) NaCl. Hydrolysis of Tweens 20 and 60 and aesculin is variable. The major cellular fatty acids are those reported previously in the species description plus iso-C_15:0_ 2-OH and anteiso-C_15:0_ 2-OH. Polar lipids are phosphatidylethanolamine, phosphatidylcholine, and several unidentified lipids. The DNA G+C range is 48.4–48.5 mol% (genome).

The type strain is KD51^T^ (= CGMCC 1.12432^T^= KCTC 32253^T^), isolated from aggregates of *Chlorella autotrophica* in Xiamen, China. The DNA G+C content of the type strain is 48.5 mol% (calculated from the genome sequence).

The species includes *Cyclobacterium halophilum* Shahinpei et al. 2014, which is a heterotypic synonym of *Cyclobacterium xiamenense*.

## Figures and Tables

**Table 1:** General features of the genomes of the type strains of species of the genus *Cyclobacterium*.

Feature	1	2	3	4	5	6	7	8
**Size** (**bp**)	6,169,285	6,158,829	5,662,104	5,792,371	5,675,162	6,221,270	6,291,928	5,784,474
**Contigs**	37	1	30	41	31	1	202	98
**Genome coverage**	193X	101X	175X	100X	247X	30X	240X	100X
**G+C** (**mol%**)	43.0	38.3	48.4	44.0	45.5	38.1	38.8	48.5
**N50** (**bp**)	547,880	6,158,829	350,204	381,560	266,214	6,221,273	107,474	137,064
**Total genes**	4943	4833	4689	4646	4736	4981	5997	4595
**Protein coding genes**	4818	4715	4635	4534	4687	4868	5958	4474
**rRNA**	6	12	5	7	5	9	4	7
**tRNA**	41	39	38	40	38	39	35	39
**Accession number**	JAANYN000000000	CP012040	FNZH00000000	WMCD00000000	FRCY00000000	NC_015914	ATNM00000000	WIOK00000000

Strains: 1, Strain GBPx2^T^; 2, *Cyclobacterium amurskyense* KCTC 12363^T^; 3, *Cyclobacterium halophilum* IBRC-M 10761^T^; 4, *Cyclobacterium jeungdinense* KCTC 23150^T^; 5, *Cyclobacterium lianum* CGMCC 1.6102^T^; 6, *Cyclobacterium marinum* DSM 745^T^; 7, *Cyclobacterium qasimii* M12-11B^T^; 8, *Cyclobacterium xiamenense* CGMCC 1.12432^T^.

**Table 2:**
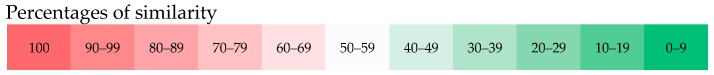
Percentages of GGDC and OrthoANI between strain GBPx2^T^ and members of the family *Cyclobacteriaceae.*

	**OrthoANI**																				
**GGDC**		**1**	**2**	**3**	**4**	**5**	**6**	**7**	**8**	**9**	**10**	**11**	**12**	**13**	**14**	**15**	**16**	**17**	**18**	**19**	**20**
	**1**	**100**	78.1	80.0	71.8	71.4	71.2	70.6	70.5	69.3	68.5	68.5	68.2	69.7	68.8	68.7	69.0	68.4	67.8	69.1	67.8
	**2**	26.9	**100**	82.4	72.0	71.1	71.4	70.6	70.5	68.8	68.1	68.3	68.4	69.6	68.6	68.7	69.0	68.6	67.7	68.9	67.6
	**3**	31.3	44.4	**100**	72.1	71.1	71.5	70.7	70.6	69.0	68.2	68.2	68.1	69.3	68.8	68.7	69.0	68.6	67.6	69.1	67.6
	**4**	14.3	14.6	14.5	**100**	79.2	76.0	74.3	74.1	69.9	69.1	68.4	67.7	69.0	69.0	68.7	68.7	68.9	68.0	69.0	67.5
	**5**	14.1	13.8	14.1	35.0	**100**	74.1	73.5	73.4	69.6	69.2	68.0	67.4	69.3	68.6	68.2	68.4	68.5	67.9	68.9	67.2
	**6**	14.2	14.3	14.4	20.8	17.8	**100**	76.6	76.4	69.3	69.2	68.0	67.5	68.6	68.8	68.3	68.2	68.7	67.9	68.7	67.3
	**7**	13.9	13.9	14	17.4	16.9	24.2	**100**	97.8	69.4	69.3	68.1	67.1	68.1	68.3	67.9	67.9	68.2	68.0	68.5	67.6
	**8**	14.0	13.9	14.1	17.4	16.7	23.9	81.6	**100**	69.2	69.2	68.0	67.1	68.3	68.2	68.1	68.2	68.1	67.7	68.7	67.6
	**9**	12.9	12.8	12.8	13	13	12.9	12.9	12.9	**100**	69.6	67.3	67.2	68.5	68.5	68.6	68.4	68.7	67.7	69.0	67.1
	**10**	12.9	12.8	12.9	12.9	12.8	12.9	13.1	13.0	13.1	**100**	68.0	67.4	68.8	68.3	68.9	68.3	69.2	68.1	68.9	67.8
	**11**	12.7	12.8	12.8	12.7	12.7	12.7	12.7	12.7	12.7	12.7	**100**	68.7	69.5	68.6	68.9	69.0	69.0	68.3	69.0	68.1
	**12**	12.7	12.7	12.7	12.6	12.5	12.6	12.6	12.6	12.6	12.6	12.7	**100**	69.7	69.0	68.7	68.9	68.6	68.0	69.0	67.5
	**13**	12.8	12.8	12.9	12.8	12.9	12.8	12.7	12.7	12.8	12.8	12.8	13.0	**100**	71.7	72.5	71.7	71.5	69.7	72.4	69.3
	**14**	12.8	12.8	12.8	12.7	12.7	12.7	12.7	12.7	12.7	12.7	12.8	12.8	13.6	**100**	70.9	70.7	70.8	70.0	71.7	68.1
	**15**	12.8	12.8	12.8	12.8	12.8	12.7	12.8	12.8	12.7	12.9	12.7	12.8	14.1	13.4	**100**	75.7	72.7	70.1	72.5	68.4
	**16**	12.8	12.7	12.8	12.7	12.7	12.7	12.8	12.8	12.7	12.7	12.9	12.8	13.6	13.2	21.2	**100**	72.2	69.9	72.4	68.2
	**17**	12.7	12.7	12.7	12.7	12.7	12.8	12.7	12.7	12.8	12.8	12.9	12.7	13.4	13.2	14.5	14.1	**100**	70.6	72.5	69.1
	**18**	12.7	12.7	12.6	12.7	12.6	12.7	12.7	12.6	12.7	12.7	12.8	12.7	12.9	12.9	13.1	13.2	12.8	**100**	70.9	68.1
	**19**	12.8	12.7	12.7	12.8	12.9	12.7	12.7	12.7	12.8	12.8	12.8	12.7	13.6	13.2	13.8	13.8	13.9	13.3	**100**	68.4
	**20**	12.7	12.7	12.7	12.7	12.6	12.7	12.7	12.7	12.6	12.7	12.8	12.7	13.1	12.8	12.8	12.8	13.1	12.7	12.8	**100**

Strains: 1, *Cyclobacterium marinum* DSM 745^T^; 2, *Cyclobacterium qasimii* M12-11B^T^; 3, *Cyclobacterium amurskyense* KCTC 12363^T^; 4, Strain GBPx2^T^; 5, *Cyclobacterium lianum* CGMCC 1.6102^T^; 6, *Cyclobacterium jeungdinens*e KCTC 23150^T^; 7, *Cyclobacterium xiamenense* CGMCC 1.12432^T^; 8, *Cyclobacterium halophilum* IBRC-M 10761^T^; 9, *Pleomorphovibrio marinus* SW125^T^; 10, *Lunatimonas lonarensis* AK24^T^; 11, *Echinicola pacifica* DSM 19836^T^; 12, *Algoriphagus ratkowskyi* DSM 22686^T^; 13, *Belliella baltica* DSM 15883^T^; 14, *Rhodonellum psychrophilum* GCM71^T^; 15, *Indibacter alkaliphilus* LW1^T^; 16, *Mongoliibacter ruber* DSM 27929^T^; 17, *Cecembia lonarensis* LW9^T^; 18, *Mariniradius saccharolyticus* AK6^T^; 19, *Aquiflexum balticum* DSM 16537^T^; 20, *Nitritalea halalkaliphila* LW7^T^. ANI: average nucleotide identity.

**Table 3:** Percentages of amino acid identity (AAI) between strain GBPx2^T^ and members of the family *Cyclobacteriaceae.*

**1**	**100**																Percentages of similarity			
**2**	83.5	**100**																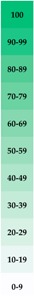		
**3**	84.7	87.5	**100**																	
**4**	73.1	73.2	73.5	**100**																
**5**	73.2	72.8	73.1	84.6	**100**															
**6**	72.9	73.1	73.5	79.5	78.0	**100**														
**7**	72.3	72.3	72.6	78.2	77.3	82.4	**100**													
**8**	72.2	72.4	72.7	78.2	77.2	82.3	97.9	**100**												
**9**	66.2	66.1	66.1	68.1	67.8	67.8	67.6	67.7	**100**											
**10**	66.0	66.1	66.2	67.3	67.5	67.4	67.5	67.6	69.1	**100**										
**11**	63.1	63.1	63.3	63.3	63.5	63.6	63.3	63.4	63.4	63.4	**100**									
**12**	61.9	61.8	61.8	61.8	61.6	61.6	61.8	61.8	61.5	61.9	63.7	**100**								
**13**	64.7	64.5	65.1	65.2	65.4	65.2	64.9	64.8	65.5	65.7	66.7	66.0	**100**							
**14**	64.0	64.1	64.1	64.7	64.4	64.7	64.3	64.5	64.6	64.8	65.7	65.4	73.2	**100**						
**15**	63.4	63.4	63.6	64.0	64.0	64.2	64.0	64.1	64.6	65.1	65.0	64.4	72.7	70.8	**100**					
**16**	63.3	63.5	63.5	63.9	63.9	64.0	63.9	64.1	64.6	64.7	65.1	64.6	72.1	70.6	81.4	**100**				
**17**	64.1	64.0	63.9	64.3	64.3	64.4	64.1	64.4	64.6	65.4	65.6	65.1	72.2	71.1	74.7	73.9	**100**			
**18**	63.2	63.1	63.1	63.6	63.4	63.9	63.6	63.6	63.7	64.5	64.6	65.1	70.5	70.1	70.6	70.8	71.9	**100**		
**19**	63.6	63.5	63.6	64.2	64.3	64.2	64.1	64.2	64.5	64.8	64.7	64.9	72.8	72.2	73.0	72.9	73.9	72.5	**100**	
**20**	62.2	62.4	62.2	62.7	62.7	62.8	63.0	63.0	62.5	63.4	63.5	63.2	66.6	65.1	66.2	65.9	66.9	65.0	65.4	**100**

Strains: 1, *Cyclobacterium marinum* DSM 745^T^; 2, *Cyclobacterium qasimii* M12-11B^T^; 3, *Cyclobacterium amurskyense* KCTC 12363^T^; 4, Strain GBPx2^T^; 5, *Cyclobacterium lianum* CGMCC 1.6102^T^; 6, *Cyclobacterium jeungdinense* KCTC 23150^T^; 7, *Cyclobacterium xiamenense* CGMCC 1.12432^T^; 8, *Cyclobacterium halophilum* IBRC-M 10761^T^; 9, *Pleomorphovibrio marinus* SW125^T^; 10, *Lunatimonas lonarensis* AK24^T^; 11, *Echinicola pacifica* DSM 19836^T^; 12, *Algoriphagus ratkowskyi* DSM 22686^T^; 13, *Belliella baltica* DSM 15883^T^; 14, *Rhodonellum psychrophilum* GCM71^T^; 15, *Indibacter alkaliphilus* LW1^T^; 16, *Mongoliibacter ruber* DSM 27929^T^; 17, *Cecembia lonarensis* LW9^T^; 18, *Mariniradius saccharolyticus* AK6^T^; 19, *Aquiflexum balticum* DSM 16537^T^; 20, *Nitritalea halalkaliphila* LW7^T^.

**Table 4:** Differential characteristics between strain GBPx2^T^ and phylogenetically related species of the genus *Cyclobacterium.* Strains: 1, strain GBPx2^T^; 2, *Cyclobacterium lianum* IBRC-M 10422^T^; 3, *Cyclobacterium jeungdonense* IBRC-M 11102^T^; 4, *Cyclobacterium halophilum* IBRC-M 10761^T^; 5, *Cyclobacterium xiamenense* CGMCC 1.12432^T^.

Characteristic	1	2	3	4	5 *
Cell size (µm)					
Outer diameter-length	0.8–1.9	1.5–1.8	1.5–1.8	0.8–1.7	1.5–2.0
Width	0.3–0.5	0.4–0.5	0.3–0.5	0.4–0.6	0.4–0.6
Salinity range (% [w/v] NaCl)	3–10	0.1–12	0–7	1–10	3–9
Growth temperature (°C):					
Range	4–40	15–40	15–35	4–35	4–40
Optimum	25	30	25	25	28
pH growth range	6.5–9.0	6.5–9.0	7.0–8.0	6.0–9.0	6.0–10.0
Nitrate reduction	+	-	+	-	-
Hydrolysis of:					
Aesculin	+	+	+	-	+
Tween 20	-	+	-	-	+
Acid production from:					
D-Arabinose	-	+	-	-	ND
D-Glucose	-	+	+	+	ND
Starch	-	+	+	-	ND
D-Xylose	-	+	-	-	ND
Utilization of:					
Cellobiose	+	+	-	-	ND
D-Mannose	+	+	-	-	w
*myo*-Inositol	+	-	-	-	-
L-Glutamic acid	-	+	-	-	ND
L-alanine	+	-	-	+	ND

DNA G+C content (mol%)†	43.0	45.4	45.6	48.4	48.5

+, Positive; -, negative; w, weak; ND, not determined. †Data for the DNA G+C content of strain GBPx2^T^ and the reference species were obtained from their genomes. * Data from Chen et al. [5].

**Table 5:** Cellular fatty acid composition (%) of strain GBPx2^T^ and related species of the genus *Cyclobacterium.* Strains: 1, GBPx2^T^; 2, *Cyclobacterium lianum* IBRC-M 10422^T^; 3, *Cyclobacterium jeungdonense* IBRC-M 11102^T^. All strains were grown under the same conditions (Marine agar medium, 25 °C, and 2 days of incubation). Fatty acids accounting for < 1% of the total content in the strains are omitted. Summed feature 3 comprised iso-C_15:0_ 2-OH and/or C_16:1_*ω7c* and summed feature 4 comprised anteiso-C_17:1_ B and/or iso-C_17:1_ I.

Fatty Acid	1	2	3
iso-C_15:1_ G	1.0	-	2.7
iso-C_15:0_	26.3	29.1	34.6
anteiso-C_15:0_	12.1	9.9	8.8
C_16:1_*ω5c*	-	5.3	2.3
iso-C_15:0_ 3-OH	2.7	4.2	1.6
iso-C_17:1_*ω9c*	9.6	8.1	12.3
C_17:1_*ω6c*	1.2	2.1	3.8
C_16:0_ 3-OH	1.0	1.2	-
iso-C_17:0_ 3-OH	12.5	10.3	7.4
C_17:0_ 2-OH	3.6	1.0	4.2
Summed feature 3	23.9	24.5	17.7
Summed feature 4	3.4	2.1	3.0

## Supplementary Materials

The following are available online at https://www.mdpi.com/2076-2607/8/4/610/s1, Figure S1: Polar lipids of strain GBPx2^T^ after two-dimensional TLC and detection with molybdophosphoric acid and heating at 200 °C for 10 min.
