# Supplementary material for: Taxogenomics of the Genus Cyclobacterium: Cyclobacterium xiamenense and Cyclobacterium halophilum as Synonyms and Description of Cyclobacterium plantarum sp. nov
Source: Microorganisms. 2020 Apr 23;8(4):610. doi: 10.3390/microorganisms8040610 (PMC7232363; doi:10.3390/microorganisms8040610)
Supplement: Supplementary file 1 [file microorganisms-08-00610-s001.pdf]

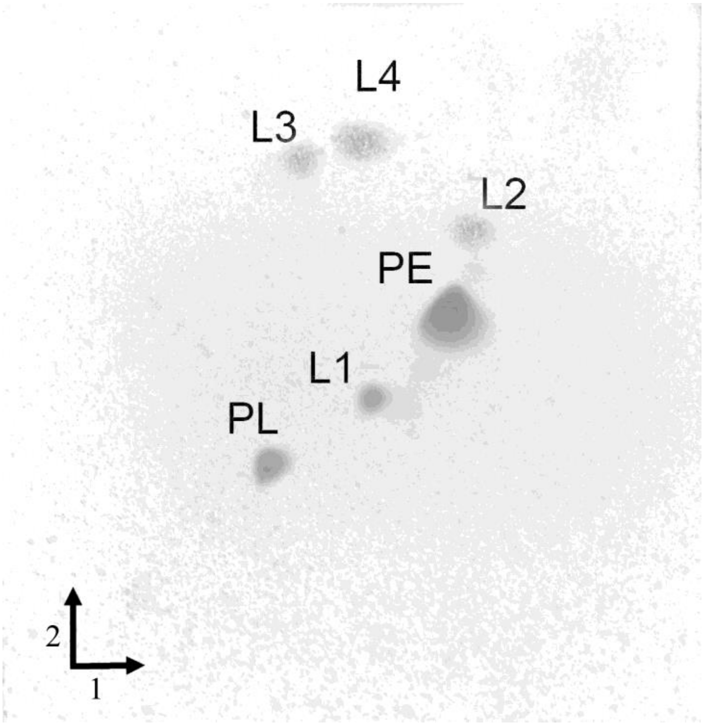

Figure S1: Polar lipids of strain GBPx2T after two-dimensional TLC and detection with molybdophosphoric acid and heating at 200 ºC for 10 min. PE, phosphatidylethanolamine; PL, unidentified phospholipid; L1–L4, unidentified lipids.
